# Energy poverty: an overlooked determinant of health and climate resilience in Canada

**DOI:** 10.17269/s41997-023-00741-0

**Published:** 2023-02-08

**Authors:** Mylène Riva, Sophie Kingunza Makasi, Kimberley C. O’Sullivan, Runa R. Das, Philippe Dufresne, David Kaiser, Sébastien Breau

**Affiliations:** 1 Department of Geography, McGill University, Montreal, Canada; 2 Department of Public Health, University of Otago, Wellington, New Zealand; 3 College of Interdisciplinary Studies, Royal Roads University, Victoria, Canada; 4 Direction régionale de santé publique de Montréal, Montreal, Canada

**Keywords:** Energy poverty, Health, Housing, Climate change, Energy transition, Précarité énergétique, santé, habitation, changements climatiques, transition énergétique

## Abstract

**Abstract:**

**Objective:**

Despite Canada being an important energy producer, not all Canadians can access or afford adequate levels of energy services at home to meet their needs, maintain healthy indoor temperatures, and live a decent life—a situation known as energy poverty. Depending on the measure, 6–19% of Canadian households face energy poverty. Health risks associated with energy poverty are documented in countries with milder climates. This study explores, for the first time in the Canadian context, the association between energy poverty and health.

**Methods:**

Cross-sectional data are from the 2018 Canadian Housing Survey. Analyses are conducted on a sample weighted to represent 14 million Canadian households. The associations between expenditure-based and self-reported measures of energy poverty and self-rated general and mental health were assessed using logistic regression models, adjusted for potential confounding variables.

**Results:**

The odds of rating one’s general (OR: 1.48; 95%CI: 1.29, 1.70) and mental (OR: 1.21; 1.04, 1.41) health as poor are significantly higher for Canadian adults in households with a high share of energy expenditure to income. The likelihood of poor general and mental health was significantly higher for those dissatisfied with the energy efficiency of their dwelling, and with their ability to maintain a comfortable temperature both in the winter and in the summer.

**Conclusion:**

Exposure to energy poverty is associated with significantly increased likelihood of poor general and mental health. Given the high proportion of Canadian households facing energy poverty, with demonstrated implications for population health, tackling energy poverty is essential for an equitable energy transition and for climate resilience.

## Résumé

**Objectif:**

Bien que le Canada soit un important producteur d’énergie, entre 6 % et 19 % des ménages canadiens, selon la mesure retenue, sont en précarité énergétique, une situation qui survient lorsqu’un ménage n’a pas les moyens ou l’accès à des services énergétiques résidentiels adéquats pour maintenir une température ambiante confortable, répondre à ses besoins et vivre dans la dignité. Les risques socio-sanitaires associés à la précarité énergétique sont documentés dans des pays au climat tempéré. Cette étude explore, pour la première fois dans le contexte canadien, l’association entre la précarité énergétique et la santé.

**Méthodes:**

Les données transversales proviennent de l’Enquête canadienne sur le logement de 2018. Les associations entre différentes mesures de précarité énergétique (mesures basées sur les dépenses des ménages et auto-rapportées) et la santé générale et mentale perçue sont estimées à l’aide de modèles de régression logistique ajustés pour des variables de confusion potentielles. Les analyses sont réalisées sur un échantillon pondéré pour représenter 14 millions de ménages.

**Résultats:**

Les probabilités de déclarer une mauvaise santé générale (OR : 1,48; IC95% : 1,29-1,70) et mentale (OR : 1,21; 1,04-1,41) sont significativement plus élevées pour les adultes canadiens dont le ménage consacre une part importante de son revenu aux coûts énergétiques. Elles sont aussi significativement plus élevées pour ceux qui déclarent être insatisfaits avec l’efficacité énergétique de leur logement et de leur capacité à maintenir une température confortable en hiver et en été.

**Conclusion:**

Vivre en situation de précarité énergétique est associée à des probabilités accrues de déclarer une mauvaise santé générale et mentale chez les adultes canadiens. En raison de la proportion élevée de ménages canadiens confrontés à la précarité énergétique et des effets socio-sanitaires que cette situation engendre, lutter contre la précarité énergétique est essentiel pour une transition énergétique équitable et pour la résilience climatique.

## Introduction

Energy is essential for meeting our basic needs and is a prerequisite for good health. Energy use is an important public health concern, as recognized by the World Health Organization in its *Housing and Health Guidelines* (World Health Organization, 2018). In Canada, home heating during the winter months is a matter of life and death (Tardy & Lee, 2019), as is home cooling during the summer. Excess mortality during heat waves, which is well documented in the Canadian context (BC Coroners Service, 2022; Lamothe et al., 2019; Lebel et al., 2019), is projected to increase over the next decades as average summer temperatures and the number of heat waves increase under likely climate scenarios.

Despite Canada being one of the largest energy producers in the world, not all Canadians can attain or afford adequate levels of energy services at home to meet their needs, maintain healthy indoor temperatures, and live a decent life—a situation known as energy poverty (Bouzarovski & Petrova, 2015; Thomson et al., 2017a; Simcock et al., 2016). Indeed, depending on the indicator, some 6% to 19% of Canadian households face energy poverty (Riva et al., 2021). Energy poverty is a complex and multidimensional phenomenon that emerges from a combination of factors, such as household income and the practices and needs of household members; the type, conditions, and energy efficiency of the dwelling; economic and political factors such as energy tariffs, available energy sources, and governance; and climate-related hazards and events that can increase or compromise energy needs (Bouzarovski et al., 2021; Hernandez, 2016; Middlemiss, 2019).

Reviews of international studies have consistently shown that exposure to energy poverty, mostly in relation to cold indoor temperature, is associated with increased risk of cardiovascular and respiratory diseases, hospitalizations, and mortality; an exacerbation of some chronic diseases; and poorer general and mental health (Jessel et al., 2019; Liddell & Morris, 2010; Marmot Review Team, 2011; O’Sullivan, 2019; World Health Organization, 2018). These associations are robust to controlling for socioeconomic characteristics and other housing conditions. In these studies, energy poverty is often measured using information on household expenditures, with households considered in energy poverty if they spend more than 10% of their annual income on home energy expenditures (10% threshold), or if the ratio of energy expenditure to household income is more than twice the national median ratio (i.e., the high share of energy expenditure to income measure, hereafter referred to as the ‘2M’ threshold). Energy poverty is also frequently measured using occupants’ self-reported assessment of the thermal comfort of their dwelling (e.g., ability to maintain comfortable indoor temperature) and energy-related financial hardships (e.g., difficulties paying for utilities; late payment or disconnection notices or disconnection for nonpayment from energy suppliers). Because each provides a partial assessment and taps into different dimensions of energy poverty, researchers have argued in favour of using both expenditure-based and self-reported measures when examining the health impacts of exposure to energy poverty (Kahouli, 2020; Llorca et al., 2020).

A comparative study of 32 European countries showed that, for most countries, greater odds of poorer self-rated health, poorer emotional well-being, and depressive symptoms were observed for those in households unable to afford keeping their dwelling warm (Thomson et al., 2017b). Among the 20 countries where a statistically significant association was observed, the odds of rating one’s health as poor when in energy poverty ranged from 1.63 (95%CI: 1.17, 2.27) in Poland to 2.80 (95%CI: 1.48, 5.29) in Spain (odds ratios were even higher for some countries, but the large confidence intervals warrant caution). In France, a study reported an 11–13% decrease in good self-rated health in association with a 10% increase in energy poverty as measured using the 10% threshold and self-reported difficulties in heating the dwelling, respectively (Kahouli, 2020). Other studies have documented associations between energy poverty and poorer general and mental health among adults (Lacroix & Chaton, 2015; Oliveras et al., 2020) and children (Oliveras et al., 2021). In Australia, results from a recent panel study showed a strong negative association between energy poverty and self-rated health in the overall sample (Churchill & Smyth, 2021). The association was weaker for middle-aged adults and for those with a university degree, whereas a stronger association was observed for those in households with children. This suggests a social pattern in the health impacts of energy poverty (Bosch et al., 2019; Mohan, 2022).

Exposure to energy poverty in the context of heatwaves and the associated health risks is receiving greater attention (Jessel et al., 2019; O’Sullivan & Chisholm, 2020; Thomson et al., 2019). In the panel study by Churchill and Smyth, the adverse effect of energy poverty on self-perceived general health was greater during the summer months and slightly stronger for Australians living in warmer states (Churchill & Smyth, 2021). While access to air conditioning (AC) is associated with reduced mortality and hospitalizations (Ostro et al., 2010; Sera et al., 2020), access to AC is socially patterned (O’Neill et al., 2005). More research is needed on the interconnections between energy poverty, the need for and access to cooling, and heat-related illnesses.

### Energy poverty in Canada

While identified as a policy issue in some provinces, e.g., in Nova Scotia where energy tariffs are among the highest in the country, energy poverty is not yet on the national policy agenda. This is likely a result of provinces having jurisdiction over most energy matters and of Canada being generally considered an energy-secure country. Yet attention directed to energy poverty is increasing because of the energy transition away from fossil fuels and its distributional effects; extreme weather events brought on by climate change that can affect energy systems from production to consumption; the fluctuation of energy prices; the affordable housing crisis (Das et al., 2022a; Kantamneni & Haley, 2021); and international conflicts.

Research on energy poverty is nascent in Canada. Information needed to compute expenditure-based measures of energy poverty (i.e., expenditure on electricity and heating and household income) is only available in the Census, the Survey of Household Spending (SHS), and the Canadian Housing Survey (CHS). The CHS is the only population survey that contains self-reported measures of thermal comfort, though these are not readily comparable to those mostly used in the EU (e.g., ability to afford warmth) and the United States (e.g., financial hardship associated with energy costs). The CHS is also the only survey to contain information on both energy poverty and health.

Data from the 2016 and 2017 SHS place the prevalence of energy poverty between 6% when measured using the 10% threshold and almost 20% when measured with the 2M threshold (with the national median ratio ~3%, thus setting the threshold for the 2M at ~6%) (Das et al., 2022b; Riva et al., 2021). In rural areas and in Atlantic provinces, over 30% of households are in energy poverty as per the 2M threshold (Riva et al., 2021). The prevalence of energy poverty is socially and spatially patterned, with one-person and older households, households with lower socioeconomic status, renters, and those living in dwellings requiring major repairs more at risk of energy poverty (Das et al., 2022b; Riva et al., 2021). Many of these same groups are also the most at risk for climate-related health impacts, in particular those associated with extreme heat. While measures of energy poverty have yet to be validated specifically for the Canadian context, current data indicate that the prevalence of domestic energy poverty, as measured by the 2M threshold, is comparable to the European average (Thomson & Bouzarovski, 2018).

To date, no Canadian study has examined the health risks associated with exposure to energy poverty. Under a changing climate, with expected increase in extreme weather events such as storms, flooding, and heatwaves (IPCC, 2021), energy poverty needs to become a priority issue. Such events have the potential to compromise households’ access to, and use of, energy. Robust data regarding energy poverty and its impacts on population health are needed to inform ongoing health-related adaptation and mitigation efforts, as well as emergency responses. This study uses microdata from the 2018 CHS to analyze the association between energy poverty and poorer self-rated general and mental health in a representative sample of Canadian adults.

## Methods

### Data

The CHS is a bi-annual survey collecting information on housing needs and housing experiences of Canadian households. A joint initiative between the Canada Mortgage and Housing Corporation and Statistics Canada, it was conducted for the first time in 2018. The target population consists of private households in the ten provinces and in selected communities in the territories. Excluded from the sampling frame are people living in retirement homes; those living full time in institutions and in other types of collective dwellings; those living in First Nations (reserves); and members of the Canadian Forces living in military bases. For this study, we focus our analysis on the ten provinces. This is because the sampling of dwellings and the administration of the questionnaire differed between the territories and the provinces and also because of the difference in energy provision in the territories, where many communities are not connected to the main grid, but rather to local power stations fueled by diesel.

In the provinces, dwellings are randomly selected within geographic strata (census metropolitan area, census agglomerations, regions outside of CMAs and CAs). Then, each geographic stratum is divided into social and affordable housing dwellings (which was a focus of the 2018 CHS) and all other dwellings. Within dwellings, one person (the ‘reference person’; aged 15 years and older) is invited to complete the questionnaire. The reference person is the household member with the most knowledge of the housing situation. In cases where members share responsibility for housing decisions, one person is chosen to be the reference person. Participation in the CHS is voluntary. Data collection for the 2018 CHS was conducted from November 2018 to March 2019, with questionnaires administered either over the phone or electronically. The overall response rate was 50%. More detailed information on the design and data collection of the CHS is available elsewhere (Statistics Canada, 2020).

### Measures

#### Energy poverty

To account for the multidimensional nature of energy poverty and to provide a greater understanding of the impacts of energy poverty on health, we considered two expenditure-based measures and three self-reported variables related to the thermal comfort provided by the dwelling.

In the CHS, respondents reported their yearly payment for electricity, and for oil, gas, coal, wood, or other fuels. Information on household income and income taxes from administrative files (Individual Tax Return from the Canada Revenue Agency) is linked to the CHS by Statistics Canada (upon consent provided by respondents). We computed ratios of energy cost (sum of expenditures for electricity + fuel) to the after-tax annual household income after housing costs, since households’ capacity to pay for energy will depend on their disposable income, after housing costs. Housing costs include rent, mortgage, property taxes, and condominium fees. The computation of expenditure-based measures was restricted to respondents in households with energy costs greater than 0 (excluding those households for whom energy bills are presumably included in their rent) and lower than annual household income after tax and with an annual household income greater than $1000. Two expenditure-based measures were computed. Households were considered energy poor if they spent more than 10% of their annual income on home energy (hereafter 10%); or if they spent more than twice the national median share of home energy costs to household income (about 6%; hereafter 2M).

Participants reported their satisfaction with their ability to maintain a comfortable temperature in the winter and in the summer, and with the energy efficiency of their dwelling. These categorical variables contrast participants reporting being satisfied or very satisfied, neither satisfied nor dissatisfied, or dissatisfied or very dissatisfied. Similar thermal comfort variables have been used as subjective indicators of energy poverty in other studies and countries, particularly in Europe (Thomson et al., 2017a).

#### Health

The CHS reference person reported on their general health and their mental health. For each measure, a dichotomous variable was created contrasting participants reporting their general or mental health as excellent, very good, and good vs. those reporting their health as fair or poor.

#### Covariates

Guided by previous studies, socioeconomic, housing, and geographical variables were considered potential confounding variables in the association between energy poverty and health. Age, gender, highest educational attainment, and financial hardship were selected as socioeconomic variables. Household and dwelling characteristics included household composition, housing tenure, and repairs needed. Province of residence and urban/rural location were considered given known variation in both energy poverty and health by region and rurality in Canada (Riva et al., 2021). Participants were classified according to the population size of their area of residence (Statistics Canada, 2017). Population centres (areas with a population ≥ 1000 and a density of ≥ 400 people per km^2^) are classified as small, medium, or large. Small population centres have a population ranging between 1000 and 29,999; medium centres have a population ranging from 30,000 to 99,999; and large centres have a population of ≥ 100,000. All areas outside population centres are considered rural.

### Statistical analysis

Data analyses were performed at the McGill-Concordia Research Data Centre (RDC), a secure physical environment available to accredited researchers to access anonymized microdata for research purposes. Analyses are conducted on a sample of more than 65,000 households residing in the 10 provinces, weighted to represent over 14 million Canadian households. Descriptive results (frequencies) are estimated as counts and transformed into percentages before being released from the RDC. Estimates with a count lower than 5 or with a coefficient of variation greater than 33.3% are not released. Separate weighted logistic regressions were applied to model the association between the five indicators of energy poverty and self-rated general and mental health, adjusting for potential confounding variables. All analysis used survey and bootstrap weights (Stata/SE 17).

## Results

Descriptive statistics of the weighted sample of respondents to the 2018 CHS living in the provinces appear in Table 1. There was an equal representation of men and women. More than half were between 34 and 65 years of age; 26% were aged 65 years and older. Close to 70% had postsecondary education and were homeowners. Major repairs needed to the dwellings were reported by 7% of respondents. About 20% reported financial hardship in the year prior to the survey. Almost 30% lived alone, and 60% lived in large urban centres. Approximately 15% reported their general health to be fair or poor, whereas 11% reported fair or poor mental health.

**Table 1:** Weighted descriptive statistics of Canadian households living in the provinces, 2018 Canadian Housing Survey

Variable		% (95%CI)
Energy poverty		
Living in a household spending more than 10% of income on energy		13.8 (13.3, 14.4)
Living in a household with a high share of energy expenditure to income (2M)		18.4 (17.8, 19.0)
Satisfaction with the energy efficiency of the dwelling	Satisfied or very satisfied	62.2 (61.5, 62.9)
	Neither satisfied nor dissatisfied	21.9 (21.3, 22.5)
	Dissatisfied or very dissatisfied	15.9 (15.4, 16.4)
Satisfaction with the ability to maintain a comfortable temperature in winter	Satisfied or very satisfied	75.3 (74.7, 75.9)
	Neither satisfied nor dissatisfied	12.0 (11.5, 12.5)
	Dissatisfied or very dissatisfied	12.7 (12.3, 13.2)
Satisfaction with the ability to maintain a comfortable temperature in summer	Satisfied or very satisfied	70.9 (70.3, 71.6)
	Neither satisfied nor dissatisfied	14.5 (14.0, 15.1)
	Dissatisfied or very dissatisfied	14.5 (14.1, 15.0)
Health measures		
Fair or poor self-rated general health		14.5 (14.0, 15.0)
Fair or poor self-rated mental health		10.6 (10.2, 11.1)
Socioeconomic characteristics		
Gender	Women	49.8 (49.1, 50.4)
	Men	50.3 (49.6, 50.9)
Age group	15–34 years	18.0 (17.6, 18.4)
	35–64 years	56.1 (55.7, 56.5)
	65 years and older	25.9 (25.6, 26.2)
Education	Less than secondary school	10.2 (9.8, 10.6)
	Secondary school completed (or equivalent)	22.0 (21.4, 22.6)
	Postsecondary education (below university)	35.3 (34.6, 36.0)
	University degree completed	32.5 (31.9, 33.2)
Difficulty meeting financial needs in the last 12 months	Difficult or very difficult	22.1 (21.6, 22.7)
	Neither difficult nor easy	41.0 (40.3, 41.7)
	Easy or very easy	36.8 (36.2, 37.5)
Household and dwelling characteristics		
Housing tenure	Owner	68.5 (68.0, 69.1)
	Renter	31.5 (30.9, 32.0)
Household composition	Couple with children	25.4 (25.0, 25.8)
	Couple without children	26.9 (26.5, 27.2)
	One lone-parent family	8.0 (7.6, 8.3)
	One census family plus additional persons	7.1 (6.8, 7.4)
	One-person (not in census family)	28.7 (28.7, 28.7)
	Two or more persons not in census families	4.0 (3.8, 4.3)
Repairs needed	Only regular maintenance	70.7 (70.0, 71.3)
	Minor repairs	22.2 (21.6, 22.8)
	Major repairs	7.1 (6.8, 7.5)
Urban/rural setting	Large urban centre	60.5 (60.1, 61.0)
	Medium population centre	9.5 (9.2, 9.9)
	Small population centre	13.0 (12.6, 13.5)
	Rural areas	16.9 (16.5, 17.3)

As per the 10% and 2M thresholds, 14% and 18% of respondents were, respectively, in energy poverty. Sixteen percent, 13%, and 15% of respondents reported being dissatisfied or very dissatisfied with the energy efficiency of their dwelling and with their ability to maintain a comfortable temperature in the winter and in the summer, respectively. There is some concordance between expenditure-based and self-reported measures (Table 2). A little over 20% of respondents categorized as energy poor per the expenditure-based measures reported being dissatisfied or very dissatisfied with the energy efficiency of their dwelling. These proportions were around 17% and 12% for the dissatisfaction with the ability to maintain a comfortable temperature in the winter and in the summer, respectively.

**Table 2:** Concordance between expenditure-based and self-reported measures of energy poverty

		Expenditure-based measures: in energy poverty as per	
		10% threshold	2M threshold
Self-reported measures		% (95%CI)	% (95%CI)
Satisfaction with the energy efficiency of the dwelling	Dissatisfied or very dissatisfied	22.4 (20.7, 24.3)	21.9 (20.4, 23.5)
	Neither dissatisfied nor satisfied	19.5 (17.7, 21.4)	19.8 (18.3, 21.4)
	Satisfied or very satisfied	58.1 (55.7, 60.4)	58.3 (56.3, 60.3)
Satisfaction with the ability to maintain a comfortable temperature in winter	Dissatisfied or very dissatisfied	17.3 (15.7, 19.0)	16.4 (15.0, 17.8)
	Neither dissatisfied nor satisfied	12.3 (10.8, 14.0)	12.7 (11.4, 14.1)
	Satisfied or very satisfied	70.4 (68.2, 72.5)	71.0 (69.1, 72.8)
Satisfaction with the ability to maintain a comfortable temperature in summer	Dissatisfied or very dissatisfied	12.4 (11.0, 13.9)	12.1 (10.9, 13.4)
	Neither dissatisfied nor satisfied	13.9 (12.2, 15.7)	13.8 (12.4, 15.4)
	Satisfied or very satisfied	73.8 (71.6, 75.9)	74.1 (72.2, 75.9)

Associations between the different indicators of energy poverty and self-rated general and mental health are shown in Table 3. In model 1 adjusting for all covariates except for financial hardship, all indicators of energy poverty were significantly associated with both self-rated general and self-rated mental health. When models were further adjusted for financial hardship (model 2), the strength of the association was reduced, indicating the potential confounding role of financial hardship in the association between energy poverty and health outcomes. Yet in all but one model, the associations remained statistically significant. In results not tabulated, we also explored the confounding role of household income. Because adjusting models for household income had almost no impact on the strength of the association between energy poverty and health outcomes, and because of the potential for multicollinearity (household income is the denominator for the expenditure-based measures of energy poverty), we elected to report results for models adjusting for financial hardship.

**Table 3:** Results from weighted and adjusted logistic regression models^a^ reporting on the associations between different indicators of energy poverty and self-rated general health and self-rated mental health, 2018 Canadian Housing Survey

		Self-rated general health		Self-rated mental health	
		Model 1^b^	Model 2^c^	Model 1^b^	Model 2^c^
		OR (95%CI)	OR (95%CI)	OR (95%CI)	OR (95%CI)
Expenditure-based energy poverty measures					
10% threshold	No	1.00	1.00	1.00	1.00
	Yes	1.57 (1.35, 1.82)	1.31 (1.12, 1.53)	1.42 (1.20, 1.67)	1.15 (0.97, 1.35)
2M threshold	No	1.00	1.00	1.00	1.00
	Yes	1.75 (1.53, 2.01)	1.48 (1.29, 1.70)	1.49 (1.28, 1.73)	1.21 (1.04, 1.41)
Self-reported energy poverty measures					
Satisfaction with the energy efficiency of the dwelling	Satisfied or very satisfied	1.00	1.00	1.00	1.00
	Neither satisfied nor dissatisfied	1.38 (1.23, 1.55)	1.23 (1.09, 1.38)	1.67 (1.48, 1.90)	1.52 (1.34, 1.73)
	Dissatisfied or very dissatisfied	1.64 (1.46, 1.84)	1.36 (1.21, 1.54)	2.07 (1.82, 2.36)	1.73 (1.51, 1.97)
Satisfaction with the ability to maintain a comfortable temperature in winter	Satisfied or very satisfied	1.00	1.00	1.00	1.00
	Neither satisfied nor dissatisfied	1.40 (1.22, 1.59)	1.21 (1.05, 1.38)	1.58 (1.37, 1.83)	1.39 (1.20, 1.61)
	Dissatisfied or very dissatisfied	1.69 (1.50, 1.91)	1.37 (1.21, 1.55)	1.77 (1.55, 2.01)	1.42 (1.24, 1.63)
Satisfaction with the ability to maintain a comfortable temperature in summer	Satisfied or very satisfied	1.00	1.00	1.00	1.00
	Neither satisfied nor dissatisfied	1.62 (1.43, 1.84)	1.42 (1.24, 1.61)	1.77 (1.54, 2.03)	1.57 (1.37, 1.81)
	Dissatisfied or very dissatisfied	1.70 (1.52, 1.92)	1.44 (1.28, 1.63)	1.90 (1.69, 2.14)	1.60 (1.42, 1.81)

^a^Each energy poverty measure is modelled in a separate logistic regression. ^b^Model 1 is adjusted for age, gender, education, household composition, repairs needed, tenure, urban/rural location, and province. ^c^Model 2 is further adjusted for economic hardship

In the fully adjusted model (model 2), when considering expenditure-based measures of energy poverty, the odds of reporting poor general health were significantly higher for those in energy poverty, as defined by both the 10% (OR: 1.31; 95%CI: 1.12, 1.53) and 2M (OR: 1.48; 95%CI: 1.29, 1.70) thresholds. The odds of poor mental health were significantly higher only when energy poverty was measured using the 2M threshold (OR: 1.21; 95%CI: 1.04, 1.41).

Overall, the odds of poor self-rated health were significantly higher for those reporting dissatisfaction with the thermal comfort of their dwelling. Compared to those who were satisfied with the energy efficiency of their dwelling, the odds of poor general (OR: 1.36; 95%CI: 1.21, 1.54) and poor mental (OR: 1.73; 95%CI: 1.51, 1.97) health were significantly higher for those reporting being dissatisfied. The likelihood of poor general (OR: 1.37; 95%CI: 1.21, 1.55) and mental health (OR: 1.42; 95%CI: 1.24, 1.63) was significantly higher for those dissatisfied with their ability to maintain a comfortable temperature in the winter. Similarly, rating one’s general (OR: 1.44; 95%CI: 1.28, 1.63) and mental health (OR: 1.60; 95%CI: 1.42, 1.81) as poor was significantly more likely among those dissatisfied with their ability to maintain a comfortable temperature in the summer.

## Discussion

This study explored, for the first time in the Canadian context, the health risks associated with exposure to energy poverty. Results demonstrate a negative association between energy poverty and the health of Canadians, with those facing energy poverty being significantly more likely to report poorer general and mental health. This effect is robust to potential confounding variables, including financial hardships and poor housing conditions, two important social determinants of health which also affect a household’s vulnerability to experience energy poverty. These results support those of recent international studies demonstrating a negative association between energy poverty and self-rated general and mental health (Bosch et al., 2019; Kahouli, 2020; Lacroix & Chaton, 2015; Oliveras et al., 2020, 2021; Thomson et al., 2017b). Findings illustrate the need for a rigorous multidisciplinary and cross-sectoral research and policy agenda to address energy poverty and its impacts on the health of Canadians.

To prevent and reduce energy poverty and increase energy security across the country, programs and policies should address the main drivers of energy poverty, i.e., energy costs borne by rate-payers and end-users, dwellings’ energy efficiency, and lower incomes (Tardy & Lee, 2019), while also targeting remedial programs and policies to households and communities that are most at risk. Energy poverty is modifiable, with potential solutions shared between the public and private sectors. Given the strong links between energy poverty and climate vulnerability, tackling energy poverty is essential to climate resilience. Cross-sectoral interventions aiming to reduce energy poverty and improve energy security by targeting the energy efficiency of dwellings have been shown to improve a range of health and well-being outcomes in varied population groups (Thomson et al., 2013; Willand et al., 2015, 2020). Residential building retrofits, for example, have the dual benefit of lowering greenhouse gas emissions and providing social and economic benefits that could assist with health by way of mitigating energy poverty (Hoicka & Das, 2021; Pojar & Karásek, 2019; Sharpe et al., 2019). Programs and subsidies for housing retrofits as well as to build new energy-efficient dwellings should consider, and be assessed for, health and equity impacts as these most often target, or are most accessible to, those who are (financially) able to make changes to their dwellings.

More research is needed to assess how energy poverty can influence other health-related outcomes, such as hospitalizations for cardiovascular and respiratory conditions, excess seasonal mortality, and heat-related illnesses. This will require linking health administrative data and vital statistics to population surveys with energy poverty–related data. Such linkages are also needed to examine causal relationships between energy poverty and health with longitudinal data. Studies should also strive to assess residential energy transition programs for their health and social impacts. There is a need to adapt, develop, and validate energy poverty measures that are relevant to the Canadian context. Measuring energy poverty in Canada is currently limited by the data available. Expenditure-based measures, such as those used in our study, measure spending on energy rather than the spending required to meet energy needs. This type of measure may thus underestimate the prevalence of energy poverty because some households reduce their energy consumption to save on costs (Papada & Kaliampakos, 2020). Despite their measurement bias, self-reported measures may better capture the social dimension of energy poverty (Papada & Kaliampakos, 2020; Thomson et al., 2017a) in relation to indoor heating and cooling. Such measures should be considered for inclusion in national housing and population health surveys.

In our results, there was a trend for self-reported measures of energy poverty to be more strongly associated with self-rated mental health, while expenditure-based indicators had a slightly greater effect on self-rated general health, as observed in other studies (Hernandez & Siegel, 2019; Oliveras et al., 2020). It is possible that self-reported measures of energy poverty are closer to the psychosocial experiences of energy poverty. These experiences can include stressors such as worries about household finance and about the ability to maintain or afford adequate temperature, the fear of having debts, and the stigma of living in a cold home (Liddell & Guiney, 2015; Middlemiss & Gillard, 2015). Qualitative research is needed to better understand the lived experience of energy poverty and the complex impacts this situation has on daily living and well-being. For example, international evidence has shown that people living in dwellings that are cold, damp, and energy inefficient feel embarrassed to receive visitors, which could lead to social exclusion and reduced well-being (Longhurst & Hargreaves, 2019; Middlemiss & Gillard, 2015). To cope with energy poverty, households may limit their energy consumption, thus exposing themselves to unhealthy thermal conditions (Chard & Walker, 2016; Longhurst & Hargreaves, 2019; O’Sullivan et al., 2017). Others opt to pay energy bills over other expenses such as food (Bhattacharya et al., 2003; Harrington et al., 2005). The lived experience of energy poverty in the context of heatwaves and managing summer heat health risks at home needs to be urgently explored.

Concerning the association between energy poverty and general health, it is possible that the stronger association with expenditure-based measures captures the physical health impacts of living in cold but also in hot dwellings. Our results show a stronger association between poorer health and dissatisfaction with the ability to maintain a comfortable temperature in the summer, compared to the ability to maintain a comfortable temperature in the winter. This illustrates the importance to consider health impacts of energy poverty in relation to household’s cooling needs (Churchill & Smyth, 2021). This is especially relevant for Canada where heatwaves are expected to increase because of climate change (IPCC, 2021).

## Conclusion

Energy poverty is an overlooked determinant of health in Canada. As Canada transitions towards cleaner energy and a lower carbon economy, it will be important to ensure that energy prices remain affordable, especially for households in situations of vulnerability. It is possible that policies to advance energy transition—for example, those targeting the housing sector—will create or exacerbate social and health inequities if potential benefits are not distributed equitably across the population. That is, programs need to exist for population groups for whom they could be most beneficial (e.g., low-income, older adults, renters). Given the high proportion of Canadian households facing energy poverty, with demonstrated implications for population health, energy poverty must be part of the discussion for an equitable energy transition and for climate resilience. Additionally, as Canada will face more intense and prolonged extreme weather events such as heatwaves, storms, and floods (IPCC, 2021), public health authorities across the country should consider including energy poverty in their climate change surveillance and monitoring programs.

## Contributions to knowledge

What does this study add to existing knowledge?

- Energy poverty is an overlooked determinant of health in Canada.
- Results show that, in a large representative sample of Canadian adults, the odds of reporting poor general health and poor mental health are significantly higher for those facing energy poverty.
- This association is robust to the measure of energy poverty selected and to adjustments for confounding variables.

What are the key implications for public health interventions, practice, or policy?

- Given the high proportion of Canadian households facing energy poverty, with demonstrated implications for population health, energy poverty must be part of the discussion for a just energy transition and for climate resilience.
- As Canada will face more intense and prolonged extreme weather events such as heatwaves, storms, and floods, public health authorities across the country should consider including energy poverty in their climate change surveillance and monitoring programs.

## Data Availability

Microdata from the 2018 Canadian Housing Survey are available through Statistics Canada’s secured Research Data Centres. More information about this survey is available at: https://www23.statcan.gc.ca/imdb/p2SV.pl?Function=getSurvey&Id=793713 The link to access the Public Use Microdata File of the 2018 Canadian Housing Survey can be found here: https://www150.statcan.gc.ca/n1/pub/46-25-0001/462500012021001-eng.htm.

## Declarations

### Disclaimer

The funders had no role in the design of the study; in the collection, analyses, or interpretation of data; in the writing of the manuscript; or in the decision to publish the results. Although the research and analysis are based on data from Statistics Canada, the opinions expressed do not represent the views of Statistics Canada or the Canadian Research Data Centre Network.
